# Childhood anaesthesia and autism risk: population and murine study

**DOI:** 10.1093/braincomms/fcae325

**Published:** 2024-09-24

**Authors:** Mingyang Sun, Ningning Fu, Ting Li, Mengrong Miao, Wan-Ming Chen, Szu-Yuan Wu, Jiaqiang Zhang

**Affiliations:** Department of Anesthesiology and Perioperative Medicine, Henan Provincial People's Hospital, People's Hospital of Zhengzhou University, Henan University People’s Hospital, Zhengzhou, Henan 450003, China; Department of Anesthesiology and Perioperative Medicine, Henan Provincial People's Hospital, People's Hospital of Zhengzhou University, Henan University People’s Hospital, Zhengzhou, Henan 450003, China; Department of Anesthesiology and Perioperative Medicine, Henan Provincial People's Hospital, People's Hospital of Zhengzhou University, Henan University People’s Hospital, Zhengzhou, Henan 450003, China; Department of Anesthesiology and Perioperative Medicine, Henan Provincial People's Hospital, People's Hospital of Zhengzhou University, Henan University People’s Hospital, Zhengzhou, Henan 450003, China; Graduate Institute of Business Administration, College of Management, Fu Jen Catholic University, Taipei 242062, Taiwan; Artificial Intelligence Development Center, Fu Jen Catholic University, Taipei 242062, Taiwan; Graduate Institute of Business Administration, College of Management, Fu Jen Catholic University, Taipei 242062, Taiwan; Artificial Intelligence Development Center, Fu Jen Catholic University, Taipei 242062, Taiwan; Center for Regional Anesthesia and Pain Medicine, Wan Fang Hospital, Taipei Medical University, Taipei 110, Taiwan; Department of Food Nutrition and Health Biotechnology, College of Medical and Health Science, Asia University, Taichung 41354, Taiwan; Big Data Center, Lo-Hsu Medical Foundation, Lotung Poh-Ai Hospital, Yilan 265, Taiwan; Division of Radiation Oncology, Lo-Hsu Medical Foundation, Lotung Poh-Ai Hospital, Yilan 265, Taiwan; Department of Healthcare Administration, College of Medical and Health Science, Asia University, 500, Lioufeng Road, Wufeng, Taichung 41354, Taiwan; Department of Anesthesiology and Perioperative Medicine, Henan Provincial People's Hospital, People's Hospital of Zhengzhou University, Henan University People’s Hospital, Zhengzhou, Henan 450003, China

**Keywords:** anaesthesia, autism, childhood, cohort study, murine model

## Abstract

Early childhood exposure to general anaesthesia has been linked to potential changes in infant brain morphology and behaviour in preclinical studies, contributing to long-term behaviours associated with autism spectrum disorder. This study investigates the association between early childhood exposure to general anaesthesia and the risk of autism, using a population-based cohort study with matching for baseline characteristics and evaluates the effect of sevoflurane exposure on autism-like behaviour in mice, using the Taiwan Maternal and Child Health Database. Children aged 0–3 who received at least one exposure to general anaesthesia between 2004 and 2014 were matched 1:1 with children who were not exposed. Risk ratios and confidence intervals were used to assess the relationship between general anaesthesia and the occurrence of autism. Additionally, mice were exposed to sevoflurane for 2 h on postnatal days 5–7, and changes in behaviour related to autism were evaluated. Propensity score matching resulted in 7530 children in each group. The incidence rates (IRs) of autism were 11.26 and 6.05 per 100 000 person-years in the exposed and unexposed groups, respectively. The incidence ratio for autism following exposure to general anaesthesia was 1.86 (95% confidence interval, 1.34–2.59). In mice, sevoflurane exposure induced autism-like behaviours and led to the downregulation of high-risk autism genes, including *ARID1B*, *GABRA5*, *GABRB3*, *GRIN2B*, *SHANK3* and *SUV420H1*. Early childhood exposure to general anaesthesia is associated with an increased risk of autism. Repeated exposure to sevoflurane in mice induces autism-like behaviours, suggesting a potential link between anaesthesia and the development of autism.

## Teaser

Sun *et al*. report that early childhood exposure to general anaesthesia is associated with an increased risk of autism spectrum disorder. The adjusted hazard ratio indicates a significant correlation, while maternal age and male gender further elevate the risk, suggesting that anaesthesia may impact neurodevelopment.

## Graphical Abstract

**Graphical abstract:**
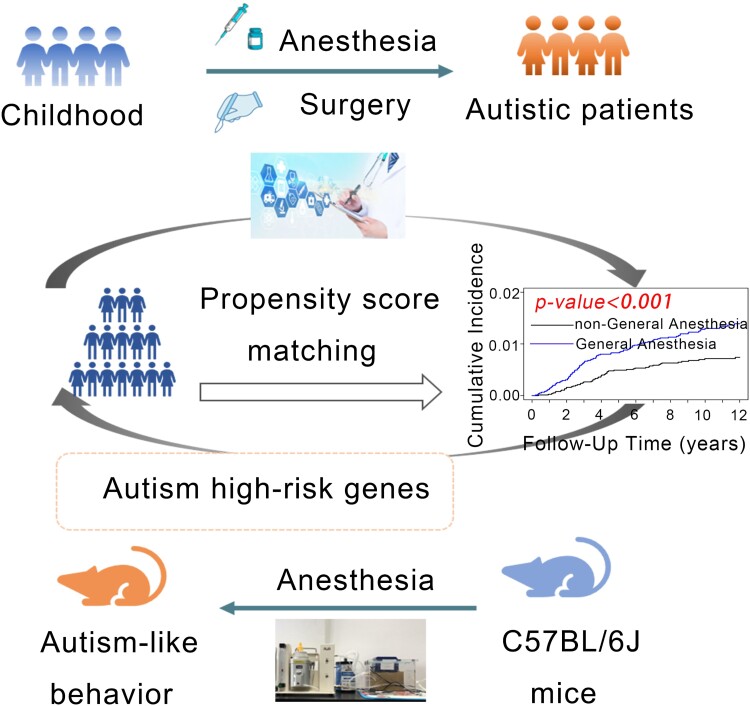

## Introduction

Autism spectrum disorder (ASD), a complex neurodevelopmental condition, manifests through social communication deficits and repetitive behaviours, with its aetiology rooted in both genetic and environmental factors. Amid rising autism incidences, understanding the role of external factors, such as anaesthesia, in ASD development becomes crucial.^1^ The spectrum encompasses a continuum of severity, ranging from mild to severe, and individuals may exhibit varying degrees of social interaction impairments, abnormal communication skills, narrow interests, stereotyped behaviours and sensory sensitivity, with intelligence levels spanning from low to high. The aetiology of ASD is multifaceted, with a prominent role played by the interplay between genetic and environmental factors. De novo mutations are recognized as significant contributors to the pathogenesis of autism, and recent attention has been directed toward exploring the epigenetic perspective. Despite ongoing research efforts, the precise pathogenic mechanisms underlying autism remain undefined.^2^ Globally, the reported prevalence of ASD is 62 per 10 000 individuals.^3^ Given the increasing incidence of autism, families face escalating economic and lifestyle challenges. Consequently, the imperative to identify effective treatments and preventive measures for ASD is paramount.

Despite the elusive nature of ASD causes, emerging findings suggest involvement of GABA signalling dysfunctions.^4-6^ Basic experimental results consistently demonstrate that early exposure to general anaesthesia induces behavioural changes, impacting learning and memory processes.^7-9^ Notably, anaesthetics like propofol, sevoflurane, isoflurane and ketamine have been associated with structural alterations, including neuronal apoptosis in rodent brains.^8,10-12^ Acting on *N*-methyl-D-aspartate glutamate receptors and GABA receptors, these anaesthetics may contribute to neurodegeneration in cortical areas, potentially leading to disorders like ASD. However, the current understanding of the relationship between general anaesthesia exposure and ASD remains unclear, hindered by inconsistencies in previous study conclusions.^13-18^ Several reports highlight a potential association between the type of anaesthesia used during caesarean sections and the occurrence of ASD.^16,17,19^ A recent meta-analysis also identified a small yet significant correlation between epidural analgesia and overall survival.^20^ Nevertheless, the available evidence falls short of conclusively proving that general anaesthesia exposure can cause or accelerate ASD formation. Therefore, further dedicated research is needed to unravel the intricate relationship between general anaesthesia and ASD.

ASD is a chronic and disabling disorder that may accompany life. The prognosis of ASD is positively associated with younger age of the child, positive attitude of parents, scientific method of intervention, adequate intervention intensity and negatively associated with the severity of onset. Therefore, we conducted a larger propensity score-matched study in infants and children younger than 3 years of age to compare the association between general anaesthesia exposure and suffering from ASD. Moreover, we established a mouse model of multiple sevoflurane exposures to verify whether it caused the occurrence of autism-like behaviour in mice.

## Supplementary Material

fcae325_Supplementary_Data

## Figures and Tables

**Table 1:** Cox proportional hazards regression for assessing autism Spectrum disorder risk in propensity score-matched infants and toddlers (aged 0–3) with and without general anaesthesia exposure (significant risk factors only)

	Crude HR (95% CI)		*P-*Value	Adjusted HR^a^ (95% CI)		*P*-Value
General anaesthesia (ref = no general anaesthesia)						
General anaesthesia	1.87	(1.34, 2.6)	<0.001	1.87	(1.34, 2.60)	<0.001
Sex (ref. female)						
Male	3.15	(1.93, 5.15)	<0.0001	3.24	(1.98, 5.3)	<0.0001
Maternal age (years) (ref. < 25)						
25–35	1.12	(1.04, 1.46)	<0.001	1.10	(1.06, 1.34)	<0.001
>35	1.55	(1.13, 1.95)	<0.0001	1.54	(1.18, 1.83)	<0.0001
Numbers of general anaesthesia (ref. 0)						
1	1.69	(1.32, 2.39)	<0.001	1.63	(1.40, 2.21)	<0.001
≥2	2.21	(2.12, 3.07)	<0.001	2.19	(2.00, 3.02)	<0.001

HR, hazard ratio; ref., reference.

^a^All variables listed in the table were accounted for in the adjusted analysis.

**Table 2:** Association between general anaesthesia exposure and incidence rate and incidence rate ratio of autism spectrum disorder in children

	Events	Person-years	Incidence rate 10 000 person year	Incidence rate ratio	95% CI for incidence rate ratio	*P*-Value
No general anaesthesia	54	89 309.0	6.05			
General anaesthesia	100	88 799.4	11.26	1.86	(1.34, 2.59)	<0.001

**Table 3:** Sensitivity analysis of autism spectrum disorder for infants and toddlers aged 0–3 years receiving general anaesthesia

Subpopulation or exposure general anaesthesia	No. of patients	Autism spectrum disorder			
		No. of autism spectrum disorder	aHR*	95% CI	*P*-Value
Sex					
Women	2212	15	5.09	(1.47, 17.61)	0.0102
Men	5318	85	1.68	(1.19, 2.38)	0.0034
Maternal age (years)					
<25	1235	13	1.18	(1.09, 2.17)	<0.001
25–35	4977	64	1.47	(1.22 2.16)	<0.001
>35	1318	23	2.47	(1.33, 2.91)	<0.001

No., number.

^*^All confounding factors listed in Supplementary Table 1 were appropriately adjusted for in the analysis.

**Figure 1:**
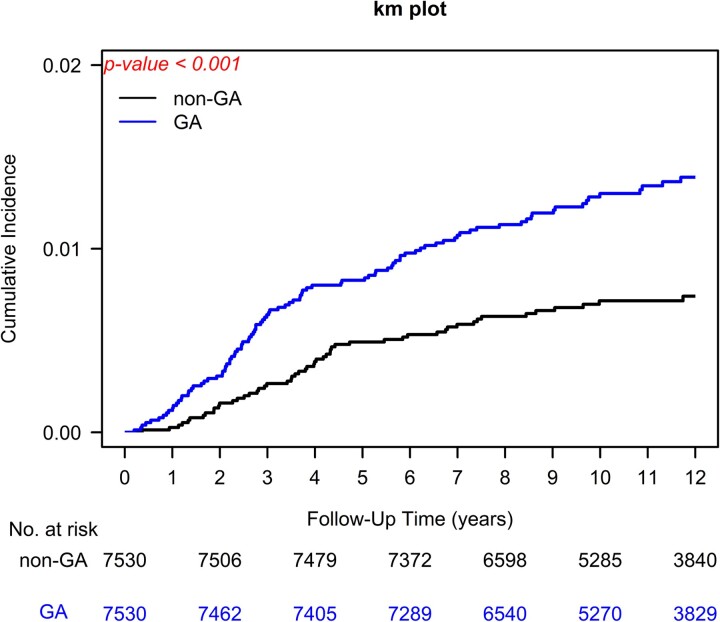
**The impact of general anaesthesia on the cumulative incidence of autism Spectrum disorder in children: a Kaplan–Meier analysis. Statistical significance was determined using a stratified log-rank test (*P* < 0.001).** GA, general anaesthesia; KM, Kaplan–Meier; No., number.

## Data Availability

The datasets supporting the study conclusions are included within this manuscript and its additional files.

## Materials and methods

### Data availability

The study's supporting data, drawn from the National Health Insurance Research Database and Taiwan Cancer Registry database, are included in the manuscript. However, access to the data is restricted due to Taiwan's Personal Information Protection Act since 2012. Unfortunately, the data cannot be publicly shared in the manuscript, supplemental files or any public repository. Interested parties must formally submit a proposal for data access, subject to approval by the relevant Taiwanese governmental department's ethics review committee. For detailed information on the data request process, please visit the following links: https://dep.mohw.gov.tw/DOS/cp-5119-59201-113.html. Informed consent was waived, as the data fall under the Personal Information Protection Act's coverage. Dr. Szu-Yuan Wu, MD, PhD, retains full access to the study data and is responsible for its integrity and accuracy. The data have not been previously presented at scientific meetings, either orally or in poster form.

### Data sources

This study utilized the Taiwan Maternal and Child Health Database (TMCHD),^21^ a comprehensive nationwide repository managed by the Health and Welfare Data Science Center in Taiwan. The TMCHD integrates diverse datasets, including Birth Registrations (2004–14), Birth Notifications (2004–14), Mortality Registrations (2004–14) and medical claims from the Taiwan National Health Insurance Research Database (2004–20). Covering a diverse population, the TMCHD ensures exceptional coverage, with a participation rate of 99%.^22-26^ Previous literature attests to the TMCHD's accuracy and thoroughness,^21,27^ and its constituent datasets provide detailed information on prenatal care, maternal health behaviours, neonatal traits and sociodemographic characteristics.^28^ The Birth Notifications dataset includes data on prenatal care, maternal health behaviours and neonatal traits, while the Birth Registrations dataset documents sociodemographic details of neonates and parents as mandated by Taiwanese legislation.^21,27,28^

### Patient selection

This study focused on individuals aged 0–3 years who underwent at least one instance of general anaesthesia and were hospitalized for over a day in Taiwan between January 2004 and December 2014. The index date was set as the general anaesthesia administration date, and the study's final follow-up date was 31 December 2021.

Our cohort study aimed to explore the potential link between early childhood exposure to general anaesthesia and ASD. ASD is a neurodevelopmental disorder characterized by persistent deficits in social communication and interaction, along with restricted, repetitive behaviour patterns. The diagnosis, based on the Diagnostic and Statistical Manual of Mental Disorders, Fifth Edition (DSM-V) criteria, includes deficits in social-emotional reciprocity, nonverbal communication and relationships, as well as restricted, repetitive behaviour patterns. Our study confirms ASD diagnoses using precise International Classification of Diseases (ICD) codes, requiring at least two or more outpatient records by a professional psychiatrist for inclusion.

Adjusting or matching for confounding factors such as maternal age, gestational age, birth weight and comorbid conditions ensures the reliability of our findings. Notably, our study excluded individuals with well-known chromosomal abnormalities associated with ASD, emphasizing the exploration of other genetic and environmental factors contributing to ASD development.

The Taiwan National Health Insurance Research Database serves as a robust resource for population-based research, offering real-world evidence for evidence-based clinical decision-making.^22-26^ The database's credibility is supported by the validation of diagnosis codes and addressing unmeasured confounders, ensuring precise disease diagnoses. Exclusions from the study encompassed individuals with pre-existing ASD, emergency surgery recipients, those who passed away during follow-up and patients diagnosed with Down syndrome or Fragile X syndrome. Propensity score matching (PSM) was employed to select eligible patients without general anaesthesia from the remaining database population.

Exclusions from the study included individuals with: (i) a documented history of ASD before or within 6 months after the index date to eliminate potential pre-existing ASD; (ii) emergency surgery; (iii) mortality during follow-up; and (iv) diagnoses of Down syndrome or Fragile X syndrome. Cases involving general anaesthesia use were identified through a database search, and 1:1 PSM was employed to randomly select eligible patients without general anaesthesia from the remaining database population.

We selected children aged 0–3 who underwent general anaesthesia from 2004 to 2014, matching them 1:1 with unexposed individuals, using the TMCHD. To examine behavioural changes, we exposed mice to 3% sevoflurane for 2 h across several days, assessing autism-related behaviours thereafter.

### PSM and covariates

After adjusting for potential confounders, we employed a time-varying Cox proportional hazards model to assess the duration between the index date and subsequent ASD diagnosis. To address variations in ASD occurrence over time after general anaesthesia, we included intervals between the index date and diagnosis as a time-varying covariate in the Cox model. Utilizing a 1:1 matching ratio with a calliper size of 0.1, we implemented PSM to balance covariates, such as age, gender, maternal age, economic status, occupational class, urbanization, parental comorbidities (ASD, depression, anxiety, drug abuse), child comorbidities (preterm birth, small for gestational age, perinatal complications, brain injury or abscess, sexual abuse, brain tumour, malnutrition, toxic exposure, infections during pregnancy, developmental delays, seizures) and medication use (anticonvulsants and sedatives) (Supplementary Table 1). Comorbidities were identified based on primary ICD-9-CM or ICD-10-CM codes in inpatient records, requiring at least two outpatient visits with the specific primary code within one year for inclusion.

### IR, IR ratio and hazard ratio of ASD in children

The study aimed to investigate the potential association between general anaesthesia exposure in infants and toddlers aged 0–3 years and the IR and IR ratio (IRR) of ASD. Using time-varying Cox regression models, adjusted hazard ratios (aHRs) with 95% confidence intervals (CIs) were estimated to assess the occurrence of ASD in both exposed and nonexposed cohorts. The primary focus was on comparing the IR and IRR of ASD, offering valuable insights into the potential risk of ASD in infants and toddlers exposed to general anaesthesia.

### Subgroup analysis

The study employed a multivariate time-varying Cox regression model for subgroup analysis, adjusting for confounding factors from Supplementary Table 1 in both anaesthesia-exposed and nonexposed cohorts. The primary goal was to explore associations between ASD and key demographic and health-related factors.

### Experimental design in animal studies

C57BL/6J mice, sourced from Gempharmatech (Nanjing, Jiangsu, China), were employed as specific pathogen-free animals at 18 days of gestation. These experimental subjects were housed in the specific pathogen-free grade animal facility at the Experimental Animal Center of Zhengzhou University, providing an environment conducive to optimal growth and development until the designated experimentation date. The housing conditions maintained a constant temperature (22 ± 2°C), relative humidity of 55–65% and a 12-h dark/12-h light cycle, with ad libitum access to food and water. Bedding changes occurred every 2–3 days according to laboratory protocols. All animal-related procedures were conducted within the time frame of 08:00–18:00. Upon reaching postnatal day 28, mice were individually caged, with no more than six mice per enclosure.

### Anaesthetic procedures in animal studies

Preceding the experiments, a pre-opened heating pad was employed to maintain a consistent temperature within the mouse anaesthesia box, ensuring the pups’ body temperature remained at 37–38°C. In accordance with prior research demonstrating the avoidance of hypoxia in a 30% oxygen environment, we utilized a gas mixture of 30% oxygen and 70% nitrogen. Mice in the sevoflurane group underwent daily inhalation of 3% sevoflurane, with concentration monitored continuously using an anaesthesia gas detector, from postnatal days 5–7 for 2 h, utilizing an oxygen flow of 1 L/min. Post-anaesthesia, pups were returned to their home cages. Control group mice inhaled a 30% oxygen concentration with an oxygen flow rate of 1 L/min and resumed their normal housing after 2 h. This anaesthesia regimen spanned 3 days, with a standardized anaesthesia time between 9 and 11 a.m. to mitigate potential confounding factors.

### Behavioural testing: open-field test

At P31, mice underwent a 1-h acclimation period in the testing room before formal experiments. Subsequently, they were introduced into a white plastic chamber (40 cm × 40 cm × 40 cm) for a 15-min session, with the initial 5 min dedicated to acclimation and the subsequent 10 min for exploration. The smart3.0 software automatically recorded the mice's exploratory behaviour during this period.

### Behavioural testing: Y-maze test

The Y-maze test, conducted at P34–35, followed a 1-h acclimation period in the testing room. The three arms of the Y-maze were labelled as the starting arm, old arm and new arm. Initially, the novel arm was enclosed by a divider, allowing the mouse to freely explore the Y-maze from the start arm for 10 min. Following a 1-h interval, the isolator was removed, positioned in the start arm facing the maze centre, and the mouse was allowed to move freely across the three arms for 5 min. During this period, the time taken and the path chosen to enter a new arm were recorded.

### Behavioural testing: marble burying test

The buried bead experiment is a commonly employed method for assessing repetitive stereotyped behaviour in animals, particularly rodents with a natural tendency to bury objects. In this experiment, a mouse cage, lined with a 5-cm thick wood shaving cushion, was utilized. Twenty glass beads (14 mm in diameter) were evenly arranged in five rows of four within the cage. Upon placing the rats into the cage, video recording ensued for 30 min, after which the rats were removed, and the number of glass beads buried (with a burial volume exceeding 2/3) was subsequently counted.

### Behavioural testing: three-chamber social test

In the sixth week of development, mice underwent the three-box social experiment, comprising three stages. The initial stage served as an adaptation period, during which the mice were placed in the central room with open doors on both sides. Identical cages were positioned in each side room, allowing the mice to freely shuttle between rooms to acclimate. The second stage evaluated social interaction tendencies, involving the introduction of a stranger (Stranger 1) into an iron cage. The tested mice were then reintroduced, and their behaviour during free activities and active contact with the unfamiliar counterpart was observed. The third stage assessed social novelty preference, wherein the tested mouse was exposed to a new stranger (Stranger 2) placed in the opposite room. Subsequent observations included the tested mouse's free movement, active contact behaviour and recorded metrics such as sniffing time per cage, time spent in each box and frequency of crossing each box.

### Behavioural testing: reciprocal social interaction test

At P42–43, the reciprocal social interaction experiment was conducted using a transparent glass tank. Before initiation, mice underwent a 10-minute adaptation period within the tank. Subsequently, they were placed back into the tank with counterparts of the same sex from different litters for interactive sessions. Video recording for 10 min documented the number and duration of interactions, encompassing behaviours such as tailing, nose-to-nose sniffing, nose-to-anus sniffing and crawling up and down each other.

### Quantitative real-time PCR

Hippocampal total RNA was extracted employing TRIzol reagent (Takara), and cDNA was synthesized with primescript Master Mix (Takara). The SYBR Green RT-PCR kit facilitated the detection of autism-related genes, with β-actin serving as the control housekeeping gene. Primer details are provided in Supplementary Table 2.

### Statistical analysis

The continuous variables of this study were expressed as means ± standard deviations, in a manner that was appropriate. To examine the connection between these variables and the risk of ASD, a time-varying Cox regression was implemented, taking into account potential clustering within matched sets using a robust sandwich estimator. Furthermore, a multivariate time-varying Cox regression analysis was conducted to estimate hazard ratios and 95% CIs and determine potential independent predictors of ASD risk. The statistical analyses were performed using SAS version 9.4 (SAS Institute, Cary, NC, USA). To assess statistical significance, a two-tailed Wald test was utilized, with a *P*-value of <0.05 deemed significant. To compare the overall IRs of ASD, Poisson regression models were employed to calculate IRRs with 95% CIs. Additionally, the cumulative incidence of ASD was evaluated through the use of the Kaplan–Meier estimator. Lastly, the incidence of ASD was compared using a stratified log-rank test, which stratified the data according to matched sets, and the results were reported as IRRs with 95% CIs.

## Results

### PSM and study cohort

This population-based paediatric cohort study employed PSM to enrol 15 060 infants and toddlers, evenly divided into two groups (*N* = 7530), as detailed in Supplementary Table 1. The PSM method ensured comparable groups with no significant differences in key variables. Despite this balance, the raw ASD IR was higher in the general anaesthesia group (1.3%) compared to the non-general anaesthesia group (0.7%), with a *P*-value of <0.001 (Supplementary Table 1).

### Predictors of ASD

The time-varying Cox proportional hazards model, a common regression technique in medical research, was employed in this study using a well-designed PSM approach.^29^ Most matched covariates showed no significant associations with ASD, except for maternal age and sex. Multivariate time-varying Cox regression analysis indicated an elevated risk of ASD in children who received general anaesthesia, with a positive correlation between the number of procedures and ASD risk. After PSM, no significant differences were found in key variables. There were no statistically significant differences regarding parental economic status, parental occupational class, urbanization, comorbidities in children or medication use, so these variables are not included in Table 1, which presents only factors with significant associations. The aHRs (95% CIs) for children undergoing general anaesthesia were 1.87 (1.34, 2.6; *P* < 0.001). The aHRs for those with one or more procedures were 1.63 (1.40, 2.21) and 2.19 (2.00, 3.02), respectively. The aHRs for maternal ages 25–35 and >35 were 1.10 (1.06, 1.34) and 1.54 (1.18, 1.83), while the aHR for males was 3.24 (1.98, 5.3) (Table 1).

### IR and IRR of ASD in general anaesthesia and no general anaesthesia groups

Table 2 reveals a significant association between the IRR of ASD and general anaesthesia. The IRs of ASD for those without and with general anaesthesia were 6.05 and 11.26 per 10 000 person-years, respectively. The IRR for the general anaesthesia group was 1.86 (95% CIs: 1.34, 2.59), indicating a higher risk of ASD in those exposed to general anaesthesia.

### Subgroup analysis

A sensitivity analysis, detailed in Table 3, explored the influence of identified risk factors from Table 1 on the anaesthesia groups to bolster the study's validity. Stratified by maternal age and gender, Cox regression models revealed results consistent with the primary analysis. Irrespective of maternal age or gender, children aged 0–3 years who underwent general anaesthesia showed an increased risk of future ASD development. These consistent findings underscore general anaesthesia as a significant risk factor for ASD in young children.

### Kaplan–Meier curves of cumulative ASD incidence

Figure 1 illustrates the cumulative incidence of ASD in each study group, revealing a significant difference (*P* < 0.001) between those who underwent general anaesthesia and those who did not, with a higher IR in the former group.

### Autism-like behaviours elicited by sevoflurane exposure

In the Y-maze test, mice subjected to multiple sevoflurane treatments exhibited reduced time spent in the novel arm compared to control mice (*P* < 0.05; Supplementary Fig. 1B). In the open-field test, the sevoflurane group demonstrated decreased entries, distance and time in the central area (*P* < 0.05; Supplementary Fig. 1C–E), indicative of sevoflurane-induced anxiety-like behaviours. The marble burying test revealed a significantly higher number of buried glass beads in the sevoflurane group compared to controls (*P* < 0.05; Supplementary Fig. 1F). In the three-chamber social test, during the Habituation stage, both control and sevoflurane groups showed no difference in exposure time to identical objects (iron cages) before and after sevoflurane anaesthesia treatment (Supplementary Fig. 2A). In the sociability stage, the time spent sniffing Stranger 1 was significantly longer than the empty side (*P* < 0.05; Supplementary Fig. 2B). However, the sevoflurane group exhibited a significantly lower time sniffing Stranger 1 than the control group (*P* < 0.05; Supplementary Fig. 2B), indicating a decreased social interaction ability following sevoflurane anaesthesia. In the social novelty stage, the control group spent significantly more time detecting Stranger 2 compared to Stranger 1 (*P* < 0.01; Supplementary Fig. 2C). In contrast, there was no significant difference in sniffing time for Stranger 1 and Stranger 2 in the sevoflurane group (Supplementary Fig. 2C), indicating the disappearance of social novelty preference in mice exposed to multiple sevoflurane treatments. The reciprocal social interaction experiment further demonstrated that the interaction time between the sevoflurane group mice and unfamiliar mice was significantly lower than that of the control group (*P* < 0.01; Supplementary Fig. 2D), confirming the induction of autism-like behaviours by multiple sevoflurane exposures in mice.

### Altered expression of autism-related genes induced by sevoflurane exposure

Following the observation of autism-like behaviours, we focused on autism high-risk genes (*ARID1B*, *GABRA5*, *GABRB3*, *GRIN2B*, *SHANK3*, *SUV420H1*)^30-34^ to investigate potential changes in gene expression induced by multiple sevoflurane exposures. As depicted in Supplementary Fig. 3, the expression of these genes was significantly downregulated after sevoflurane exposure. This underscores the crucial role these genes play in sevoflurane-induced autism-like behaviour, offering a novel avenue for exploring the molecular mechanisms underlying sevoflurane-induced neurotoxicity.

After matching, we analysed 7530 cases from each group, finding that anaesthesia-exposed children had a higher ASD incidence (11.26 versus 6.05 per 100 000 person-years). Our murine studies further showed that sevoflurane exposure led to autism-like behaviours and downregulated autism-associated genes.

## Discussion

Our comprehensive analysis reveals a significant association between general anaesthesia exposure in infants and young children and an elevated long-term risk of ASD. Following PSM, our findings revealed a notably elevated risk of ASD associated with general anaesthesia exposure. Subgroup analysis further underscored the elevated risk of ASD from general anaesthesia across all subsets, unaffected by maternal age or the gender of the infant. Notably, parallel animal experiments demonstrated that multiple sevoflurane exposures induce autism-like behaviours in mice, providing additional support for the observed clinical association.

Studies investigating the link between ASD and anaesthesia have produced inconsistent outcomes, reflecting the complexity of establishing a clear connection.^14,17-19,35^ Notably, a retrospective cohort study reported no significant association between general anaesthesia exposure and the risk of developing ASD.^17^ However, this study specifically focused on infants below 2 years of age, conducted a short-term follow-up and limited analysis to data from the years of extraction without subsequent follow-ups.^17^ In another population-based case-control study, general anaesthesia was not found to be correlated with an elevated risk of ASD.^14^ Nevertheless, the study faced limitations due to a relatively constrained number of cases, rendering it inadequate for detecting odds ratios.^14^ Furthermore, the study population exclusively represented residents of Olmsted County, thereby potentially limiting the generalizability of the findings to broader regional populations.^14^ A separate case-control study suggested a potential connection between general anaesthesia exposure and the development of ASD in individuals with a history of caesarean delivery.^19^ Noteworthy, this study primarily focused on maternal anaesthesia exposure during caesarean delivery, contributing a nuanced perspective to the existing body of literature.

Consistent findings from animal studies demonstrate that common anaesthetics may lead to social behaviour abnormalities, aligning with our observations.^2,36,37^ This alignment is further corroborated by our investigation utilizing a model of multiple sevoflurane exposures in young rats, substantiating the notion that individuals exposed to anaesthesia at an early age may face an elevated risk of ASD. However, it is crucial to acknowledge that an animal study has reported mixed results.^38^ Notably, this study employed a titrated concentration of sevoflurane, raising concerns about the ability to definitively rule out a potential association between anaesthesia exposure during the juvenile period in mice and subsequent neurodevelopmental impairment. The nuanced findings underscore the need for continued exploration and refinement of our understanding of the relationship between early anaesthesia exposure and neurodevelopmental outcomes.

Our methodologically robust study integrates clinical data with animal research to illuminate the potential risks of anaesthesia exposure on ASD. To mitigate potential biases, we implemented PSM to model a randomized controlled trial. Additionally, we conducted subgroup analyses by gender and maternal age, while concurrently adjusting for relevant covariates. This multifaceted approach allowed for a thorough exploration of the potential influence of anaesthesia on the risk of ASD, enhancing the robustness and reliability of our findings.

While our findings are compelling, it is crucial to acknowledge several limitations inherent in our study. First, our clinical experiment exclusively involved patients from the Taiwanese Asian population, introducing a potential divergence from findings in a prior study conducted in a European population. However, the absence of current evidence indicating racial disparities in ASD risk suggests caution in interpreting such discrepancies. Second, despite our implementation of PSM to balance potential confounding factors linked to ASD, unanticipated confounders during the perioperative period of anaesthesia may persist. Third, ASD diagnoses in our study relied on ICD-9 or ICD-10 codes; however, stringent criteria were applied, with inclusion limited to patients possessing two or more outpatient records from a specialist psychiatrist. Fourth, our study adhered to an observational design, underscoring the necessity for large-scale randomized controlled experiments to conclusively verify the effects of anaesthesia on ASD. Lastly, while we employed an animal model to assess anaesthesia-induced behavioural changes in mice, the study exclusively utilized sevoflurane as the anaesthetic agent, thus potentially limiting the model's representativeness for simulating anaesthesia in infants and young children, where multiple anaesthetic drugs might be employed.

## Conclusions

Our retrospective cohort analysis suggests a potential association between early-life exposure to general anaesthesia in infants and an elevated risk of ASD, irrespective of maternal age or sex. This finding is further supported by consistent results observed in our animal experiments. However, to establish a causal relationship and elucidate the underlying mechanisms, confirmation through prospective randomized controlled trials is imperative. These trials would provide a more robust foundation for informing clinical decisions and refining our understanding of the impact of early anaesthesia exposure on ASD risk.

## References

[1] Lord C, Elsabbagh M, Baird G, Veenstra-Vanderweele J. Autism spectrum disorder. Lancet. 2018;392(10146):508–520. 30078460 10.1016/S0140-6736(18)31129-2 PMC7398158

[2] Sheng Z, Liu Q, Cheng C, et al Fentanyl induces autism-like behaviours in mice by hypermethylation of the glutamate receptor gene Grin2b. Br J Anaesth. 2022;129(4):544–554. 35697546 10.1016/j.bja.2022.04.027

[3] Elsabbagh M, Divan G, Koh YJ, et al Global prevalence of autism and other pervasive developmental disorders. Autism Res. 2012;5(3):160–179. 22495912 10.1002/aur.239 PMC3763210

[4] Puts NAJ, Wodka EL, Harris AD, et al Reduced GABA and altered somatosensory function in children with autism spectrum disorder. Autism Res. 2017;10(4):608–619. 27611990 10.1002/aur.1691 PMC5344784

[5] Nakamura T, Arima-Yoshida F, Sakaue F, et al PX-RICS-deficient mice mimic autism spectrum disorder in Jacobsen syndrome through impaired GABAA receptor trafficking. Nat Commun. 2016;7:10861. 26979507 10.1038/ncomms10861 PMC4799364

[6] Chao HT, Chen H, Samaco RC, et al Dysfunction in GABA signalling mediates autism-like stereotypies and Rett syndrome phenotypes. Nature. 2010;468(7321):263–269. 21068835 10.1038/nature09582 PMC3057962

[7] Liu X, Ji J, Zhao GQ. General anesthesia affecting on developing brain: Evidence from animal to clinical research. J Anesth. 2020;34(5):765–772. 32601887 10.1007/s00540-020-02812-9 PMC7511469

[8] Fu N, Wang Y, Zhu R, et al Bicuculline and bumetanide attenuate sevoflurane-induced impairment of myelination and cognition in young mice. ACS Chem Neurosci. 2023;14(6):1146–1155. 36802490 10.1021/acschemneuro.2c00764

[9] Liang X, Zhang Y, Zhang C, et al Effect of repeated neonatal sevoflurane exposure on the learning, memory and synaptic plasticity at juvenile and adult age. Am J Transl Res. 2017;9(11):4974–4983. 29218095 PMC5714781

[10] Schenning KJ, Noguchi KK, Martin LD, et al Isoflurane exposure leads to apoptosis of neurons and oligodendrocytes in 20- and 40-day old rhesus macaques. Neurotoxicol Teratol. 2017;60:63–68. 27876652 10.1016/j.ntt.2016.11.006 PMC5367949

[11] Creeley C, Dikranian K, Dissen G, Martin L, Olney J, Brambrink A. Propofol-induced apoptosis of neurones and oligodendrocytes in fetal and neonatal rhesus macaque brain. Br J Anaesth. 2013;110(Suppl 1):i29–i38. 23722059 10.1093/bja/aet173 PMC3667347

[12] Brambrink AM, Evers AS, Avidan MS, et al Ketamine-induced neuroapoptosis in the fetal and neonatal rhesus macaque brain. Anesthesiology. 2012;116(2):372–384. 22222480 10.1097/ALN.0b013e318242b2cd PMC3433282

[13] Houck P, Naus C, Croen L, Sun LS. Perinatal anesthesia exposure and autism Spectrum disorders. J Neurosurg Anesthesiol. 2023;35(1):127–129. 36745175 10.1097/ANA.0000000000000879

[14] Laporta ML, Sprung J, Fejedelem CA, et al Association between exposure of children to general anesthesia and autism spectrum disorder. J Autism Dev Disord. 2022;52(10):4301–4310. 34618293 10.1007/s10803-021-05305-0 PMC9620709

[15] Yang YL, Wang LJ, Chang JC, Ho SC, Kuo HC. A national population cohort study showed that exposure to general anesthesia in early childhood is associated with an increase in the risk of developmental delay. Children (Basel). 2021;8(10):840. 34682104 10.3390/children8100840 PMC8534755

[16] Yang Y, Lin J, Lu X, et al Anesthesia, sex and miscarriage history may influence the association between cesarean delivery and autism spectrum disorder. BMC Pediatr. 2021;21(1):62. 33522911 10.1186/s12887-021-02518-1 PMC7849114

[17] Ko WR, Huang JY, Chiang YC, et al Risk of autistic disorder after exposure to general anaesthesia and surgery: A nationwide, retrospective matched cohort study. Eur J Anaesthesiol. 2015;32(5):303–310. 25101714 10.1097/EJA.0000000000000130

[18] DiMaggio C, Sun LS, Li G. Early childhood exposure to anesthesia and risk of developmental and behavioral disorders in a sibling birth cohort. Anesth Analg. 2011;113(5):1143–1151. 21415431 10.1213/ANE.0b013e3182147f42 PMC3164160

[19] Huberman Samuel M, Meiri G, Dinstein I, et al Exposure to general anesthesia may contribute to the association between cesarean delivery and autism spectrum disorder. J Autism Dev Disord. 2019;49(8):3127–3135. 31053992 10.1007/s10803-019-04034-9

[20] Fang LL, Zhou YY, Jiang HY, Shi YD. Labor epidural analgesia and risk of autism Spectrum disorders in offspring: A systematic review and meta-analysis. Front Pediatr. 2022;10:965205. 36890990 10.3389/fped.2022.965205 PMC9986298

[21] Chou HH, Chiou MJ, Liang FW, Chen LH, Lu TH, Li CY. Association of maternal chronic disease with risk of congenital heart disease in offspring. CMAJ. 2016;188(17–18):E438–E446. 27729382 10.1503/cmaj.160061 PMC5135520

[22] Zhang J, Lu CY, Chen HM, Wu SY. Neoadjuvant chemotherapy or endocrine therapy for invasive ductal carcinoma of the breast with high hormone receptor positivity and human epidermal growth factor receptor 2 negativity. JAMA Netw Open. 2021;4(3):e211785. 33710293 10.1001/jamanetworkopen.2021.1785 PMC7955271

[23] Wu SY, Chang CL, Chen CI, Huang CC. Comparison of acute and chronic surgical complications following robot-assisted, laparoscopic, and traditional open radical prostatectomy among men in Taiwan. JAMA Netw Open. 2021;4(8):e2120156. 34432012 10.1001/jamanetworkopen.2021.20156 PMC8387846

[24] Lin KC, Chen TM, Yuan KS, Wu ATH, Wu SY. Assessment of predictive scoring system for 90-day mortality among patients with locally advanced head and neck squamous cell carcinoma who have completed concurrent chemoradiotherapy. JAMA Netw Open. 2020;3(3):e1920671. 32215631 10.1001/jamanetworkopen.2019.20671 PMC12507455

[25] Lo YL, Li MC, Yu YH, Chen HM, Wu SY. Long-term survival outcomes and prognostic factors related to ruptured intracranial aneurysms: A comparison of surgical and endovascular options in a propensity score-matched, nationwide population-based cohort study. Eur J Neurol. 2021;28(9):3012–3021. 34192398 10.1111/ene.15002

[26] Sun M, Chen WM, Wu SY, Zhang J. Dementia risk after major elective surgery based on the route of anaesthesia: A propensity score-matched population-based cohort study. EClinicalMedicine. 2023;55:101727. 36386032 10.1016/j.eclinm.2022.101727 PMC9641180

[27] Wu TP, Liang FW, Huang YL, Chen LH, Lu TH. Maternal mortality in Taiwan: A nationwide data linkage study. PLoS One. 2015;10(8):e0132547. 26237411 10.1371/journal.pone.0132547 PMC4523206

[28] Lin CM, Lee PC, Teng SW, Lu TH, Mao IF, Li CY. Validation of the Taiwan Birth Registry using obstetric records. J Formos Med Assoc. 2004;103(4):297–301. 15175826

[29] Lin DY, Wei L-J. The robust inference for the Cox proportional hazards model. J Am Stat Assoc. 1989;84(408):1074–1078.

[30] Vutskits L . Opioids and autism spectrum disorder: Liaisons dangereuses? Br J Anaesth. 2023;130(4):393–395. 36754706 10.1016/j.bja.2023.01.003

[31] Coskunpinar EM, Tur S, Cevher Binici N, Yazan Songür C. Association of GABRG3, GABRB3, HTR2A gene variants with autism spectrum disorder. Gene. 2023;870:147399. 37019319 10.1016/j.gene.2023.147399

[32] Abdel-Haq M, Ojha SK, Hamoudi W, et al Effects of extended-release 7-nitroindazole gel formulation treatment on the behavior of Shank3 mouse model of autism. Nitric Oxide. 2023;140–141:41–49. 10.1016/j.niox.2023.09.003 37714296

[33] Kim H, Kim D, Cho Y, et al Early postnatal serotonin modulation prevents adult-stage deficits in Arid1b-deficient mice through synaptic transcriptional reprogramming. Nat Commun. 2022;13(1):5051. 36030255 10.1038/s41467-022-32748-5 PMC9420115

[34] Wang ZJ, Rein B, Zhong P, et al Autism risk gene KMT5B deficiency in prefrontal cortex induces synaptic dysfunction and social deficits via alterations of DNA repair and gene transcription. Neuropsychopharmacology. 2021;46(9):1617–1626. 34007043 10.1038/s41386-021-01029-y PMC8280130

[35] Chien LN, Lin HC, Shao YH, Chiou ST, Chiou HY. Risk of autism associated with general anesthesia during cesarean delivery: A population-based birth-cohort analysis. J Autism Dev Disord. 2015;45(4):932–942. 25256350 10.1007/s10803-014-2247-y

[36] Yonamine R, Satoh Y, Kodama M, Araki Y, Kazama T. Coadministration of hydrogen gas as part of the carrier gas mixture suppresses neuronal apoptosis and subsequent behavioral deficits caused by neonatal exposure to sevoflurane in mice. Anesthesiology. 2013;118(1):105–113. 23221861 10.1097/ALN.0b013e318275146d

[37] Satomoto M, Satoh Y, Terui K, et al Neonatal exposure to sevoflurane induces abnormal social behaviors and deficits in fear conditioning in mice. Anesthesiology. 2009;110(3):628–637. 19212262 10.1097/ALN.0b013e3181974fa2

[38] Chung W, Park S, Hong J, et al Sevoflurane exposure during the neonatal period induces long-term memory impairment but not autism-like behaviors. Paediatr Anaesth. 2015;25(10):1033–1045. 26095314 10.1111/pan.12694

