# Supplementary material for: Childhood anaesthesia and autism risk: population and murine study
Source: Brain Commun. 2024 Sep 24;6(5):fcae325. doi: 10.1093/braincomms/fcae325 (PMC11450270; doi:10.1093/braincomms/fcae325)
Supplement: fcae325_Supplementary_Data [file fcae325_supplementary_data.docx]

## Supplemental Tables

Supplemental Table 1. Comparison of Propensity Score-Matched Characteristics in Infants and Toddlers Aged 0-3 Years Receiving or Not Receiving General Anesthesia

Supplemental table 2. Primers Utilized in the Experiments

## Supplemental Figure Legends

Supplemental Figure 1. Sevoflurane-Induced Autism-Like Behaviors in Mice.

(A) Experimental design. (B) Time to enter the open arm in the Y-maze experiment. (C-E) The time, number of times, and distance to enter the central area in the open field experiment. (F) Quantification of the total number of marbles buried during the marble burying test. Values are presented as mean ± SEM. *p < 0.05, **p < 0.01 (N = 10 per group), Student’s t-test.

**Footnote**: SEM, standard error of the mean; N, number of mice in each group. Statistical significance was determined using Student’s t-test (*p < 0.05, **p < 0.01).

Supplemental Figure 2. Sevoflurane-Exposed Mice Exhibit Social Interaction Disorder.

(A) During the first stage (Habituation), mice in the sevoflurane group exhibited no significant difference in the time spent sniffing the two cages. (B) In the second stage (Sociability), the time spent sniffing stranger 1 was higher than that of sniffing the empty side in both groups; however, the time spent sniffing stranger 1 was higher in the control group compared to the sevoflurane group. (C) In the third stage (Social Novelty), there was no difference in the time spent sniffing stranger 1 and stranger 2 in the sevoflurane group, indicating a disappearance of social novelty preference. (D) Total interaction time between mice in the sevoflurane group and unfamiliar mice. Values are presented as mean ± SEM. *p < 0.05, **p < 0.01, #p < 0.05 (N = 10 per group), Student’s t-test.

**Footnote**: SEM, standard error of the mean; N, number of mice in each group. Statistical significance was determined using Student’s t-test (*p < 0.05, **p < 0.01, #p < 0.05).

Supplemental Figure 3. Altered Expression of Autism-Related Genes Following Sevoflurane Anesthesia.

(A) mRNA expression of ARID1B. (B) mRNA expression of GABRA5. (C) mRNA expression of GABRB3. (D) mRNA expression of GRIN2B. (E) mRNA expression of SHANK3. (F) mRNA expression of SUV420H1. Values are presented as mean ± SEM. *p < 0.05, **p < 0.01, Student’s t-test. Each group consists of five mice.

**Footnote**: SEM, standard error of the mean. Statistical significance was determined using Student’s t-test (*p < 0.05, **p < 0.01).

**Supplemental Table 1. Comparison of Propensity Score-Matched Characteristics in Infants and Toddlers Aged 0-3 Years Receiving or Not Receiving General Anesthesia**

|  | **No General Anesthesia** | | **General Anesthesia** | | ***P*-value** |
| --- | --- | --- | --- | --- | --- |
|  | **N=7530** | | **N=7530** | |  |
|  | **N** | **%** | **N** | **%** |  |
| **Age** (mean±SD) | 1.46 ± 0.97 | | 1.46 ± 0.97 | | 1.000 |
| Age, median (IQR), years old | 1.00 (1.00,2.00) | | 1.00 (1.00,2.00) | | 1.000 |
| Age group, years-old |  |  |  |  | 1.000 |
| 0-0.5 | 1,405 | 18.7% | 1,405 | 18.7% |  |
| 0.6-1 | 2,473 | 32.8% | 2,473 | 32.8% |  |
| 1.1-2 | 2,425 | 32.2% | 2,425 | 32.2% |  |
| 2.1-3 | 1,227 | 16.3% | 1,227 | 16.3% |  |
| **Sex** |  |  |  |  | 1.000 |
| Female | 2,212 | 29.4% | 2,212 | 29.4% |  |
| Male | 5,318 | 70.6% | 5,318 | 70.6% |  |
| **Maternal age** (years-old) |  |  |  |  | 1.000 |
| <25 | 1,235 | 16.4% | 1,235 | 16.4% |  |
| 25-35 | 4,977 | 66.1% | 4,977 | 66.1% |  |
| >35 | 1,318 | 17.5% | 1,318 | 17.5% |  |
| **Parental economic status (NTD)** |  |  |  |  | 1.000 |
| Less than NT$20,000 | 2,003 | 26.6% | 2,003 | 26.6% |  |
| NT$20,000 to NT$39,999 | 3,697 | 49.1% | 3,697 | 49.1% |  |
| NT$40,000 or more | 1,830 | 24.3% | 1,830 | 24.3% |  |
| **Parental Occupational Class** |  |  |  |  | 1.000 |
| Unemployment | 43 | 0.6% | 43 | 0.6% |  |
| Scholars | 469 | 6.2% | 468 | 6.2% |  |
| Agriculture | 351 | 4.7% | 351 | 4.7% |  |
| Industry | 539 | 7.2% | 543 | 7.2% |  |
| Commerce | 2,446 | 32.5% | 2,434 | 32.3% |  |
| Financial dependent | 3,682 | 48.9% | 3,691 | 49.0% |  |
| **Urbanization** |  |  |  |  | 0.5789 |
| Rural | 1,999 | 26.6% | 1,969 | 26.2% |  |
| Urban | 5,531 | 73.5% | 5,561 | 73.9% |  |
| **Parental Comorbidities** |  |  |  |  |  |
| Autism Spectrum Disorder | 3 | 0.0% | 3 | 0.0% | 1.000 |
| Depression | 188 | 2.5% | 188 | 2.5% | 1.000 |
| Anxiety | 279 | 3.7% | 279 | 3.7% | 1.000 |
| Drug abuse | 76 | 1.0% | 76 | 1.0% | 1.000 |
| **Comorbidities in Children** |  |  |  |  |  |
| Preterm and SGA | 113 | 1.5% | 112 | 1.5% | 0.981 |
| Perinatal complications | 1,279 | 17.0% | 1,282 | 17.0% | 0.948 |
| Brain injury or Brain abscess | 1,250 | 16.6% | 1,242 | 16.5% | 0.933 |
| Sexual abuse | 23 | 0.3% | 21 | 0.3% | 0.942 |
| Brain tumor | 7 | 0.1% | 7 | 0.1% | 1.000 |
| Malnutrition | 166 | 2.1% | 167 | 2.2% | 0.964 |
| Toxic exposure | 271 | 3.6% | 273 | 3.6% | 0.963 |
| Infections during pregnancy | 60 | 0.8% | 60 | 0.8% | 1.000 |
| Developmental delays | 819 | 10.9% | 821 | 10.9% | 0.973 |
| Seizures | 22 | 0.3% | 23 | 0.3% | 0.961 |
| **Medications use** |  |  |  |  |  |
| Anticonvulsants | 8 | 0.1% | 8 | 0.1% | 1.000 |
| Sedatives | 11 | 0.1% | 11 | 0.1% | 1.000 |
| **Numbers of general anesthesia** |  |  |  |  |  |
| **0** | 7,530 | 100% | 0 | 0.0% |  |
| **1** | 0 | 0.0% | 6,419 | 85.3% |  |
| **>=2** | 0 | 0.0% | 1,111 | 14.8% |  |
| **Outcomes** |  |  |  |  |  |
| **Autism Spectrum Disorder** |  |  |  |  | <0.001 |
|  | 7,476 | 99.3% | 7,430 | 98.7% |  |
|  | 54 | 0.7% | 100 | 1.3% |  |

**Abbreviations**: SD: Standard Deviation; IQR: Interquartile Range; Q1: First Quartile; Q3: Third Quartile; N: Sample Size; SGA: Small for Gestational Age.

**Supplemental table 2.** Primers Utilized in the Experiments

| **Genes** | **Sequence (5′-greater than3′)** |
| --- | --- |
| *β-actin* | FW: GGCTGTATTCCCCTCCATCG  RE: CCAGTTGGTAACAATGCCATGT |
| *SUV420H1* | FW: CGGCTGCTTCCAACTCTACC  RE: AGTGATTCCGCAGTCTGATCT |
| *ARID1B*  *GABRA5*  *GRIN2B*  *GABRB3*  *SHANK3* | FW: CGTGCGGAGCTTGTCTTTC  RE: CCTCCTTCTCATAGGTCTGTGG  FW: TGACCCAAACCCTCCTTGTCT  RE: TGACCCAAACCCTCCTTGTCT  FW: TCCGCCGTGAGTCTTCTGTCTATG  RE: CTGGGTGGTAAAGGGTGGGTTGTC  FW: CTGCTGCCAATCTGGCTTTC  RE: CGTAGCCTTTCAACAGCTTGTC  FW: ATGGGCCTGTGTGGTAGTCTT  RE: CCACCTTATCTGTGCTGTGTAG |

Supplemental Figure 1. Sevoflurane-Induced Autism-Like Behaviors in Mice.

(A) Experimental design. (B) Time to enter the open arm in the Y-maze experiment. (C-E) The time, number of times, and distance to enter the central area in the open field experiment. (F) Quantification of the total number of marbles buried during the marble burying test. Values are presented as mean ± SEM. *p < 0.05, **p < 0.01 (N = 10 per group), Student’s t-test.


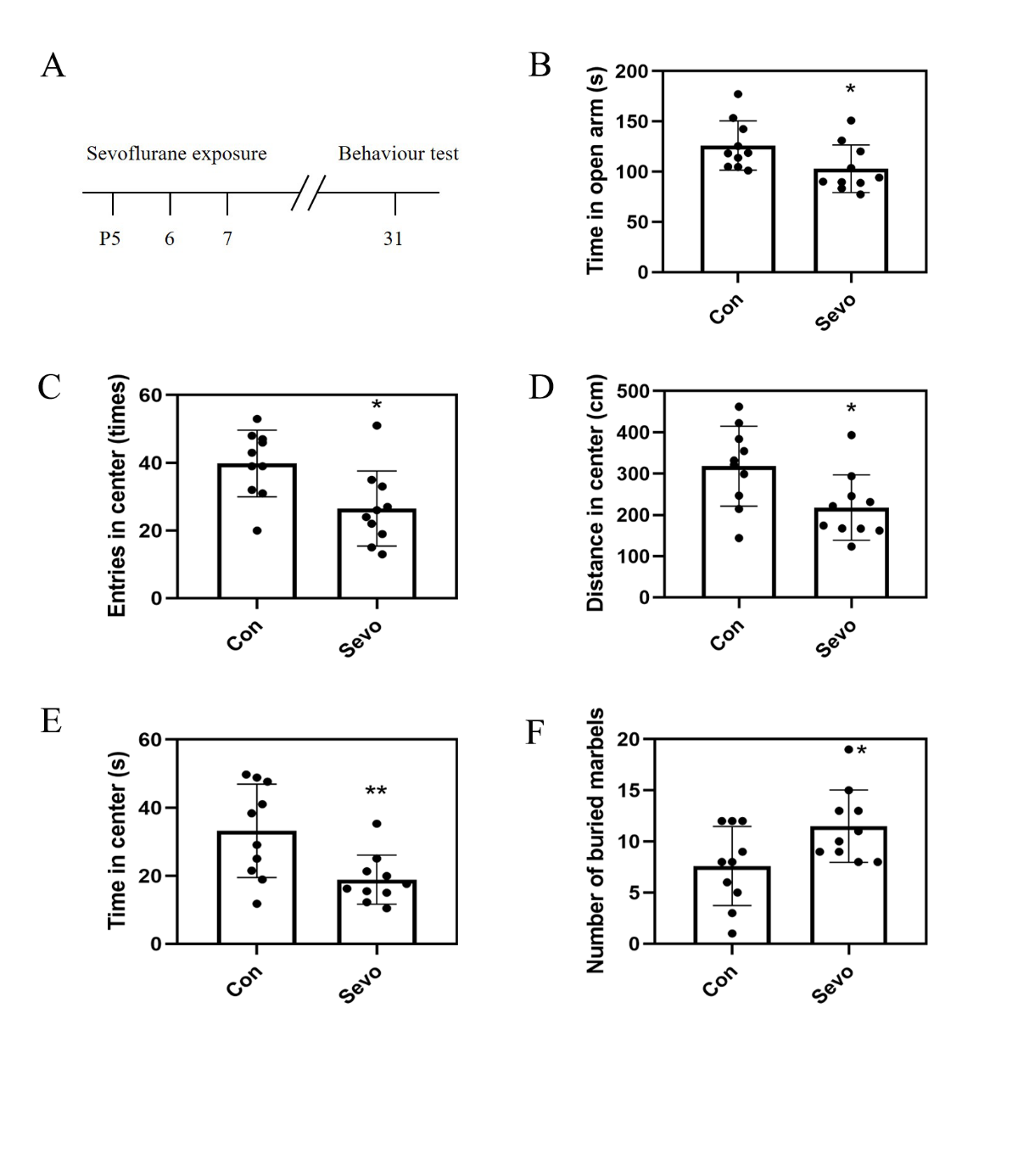


**Footnote**: SEM, standard error of the mean; N, number of mice in each group. Statistical significance was determined using Student’s t-test (*p < 0.05, **p < 0.01).

Supplemental Figure 2. Sevoflurane-Exposed Mice Exhibit Social Interaction Disorder.

(A) During the first stage (Habituation), mice in the sevoflurane group exhibited no significant difference in the time spent sniffing the two cages. (B) In the second stage (Sociability), the time spent sniffing stranger 1 was higher than that of sniffing the empty side in both groups; however, the time spent sniffing stranger 1 was higher in the control group compared to the sevoflurane group. (C) In the third stage (Social Novelty), there was no difference in the time spent sniffing stranger 1 and stranger 2 in the sevoflurane group, indicating a disappearance of social novelty preference. (D) Total interaction time between mice in the sevoflurane group and unfamiliar mice. Values are presented as mean ± SEM. *p < 0.05, **p < 0.01, #p < 0.05 (N = 10 per group), Student’s t-test.


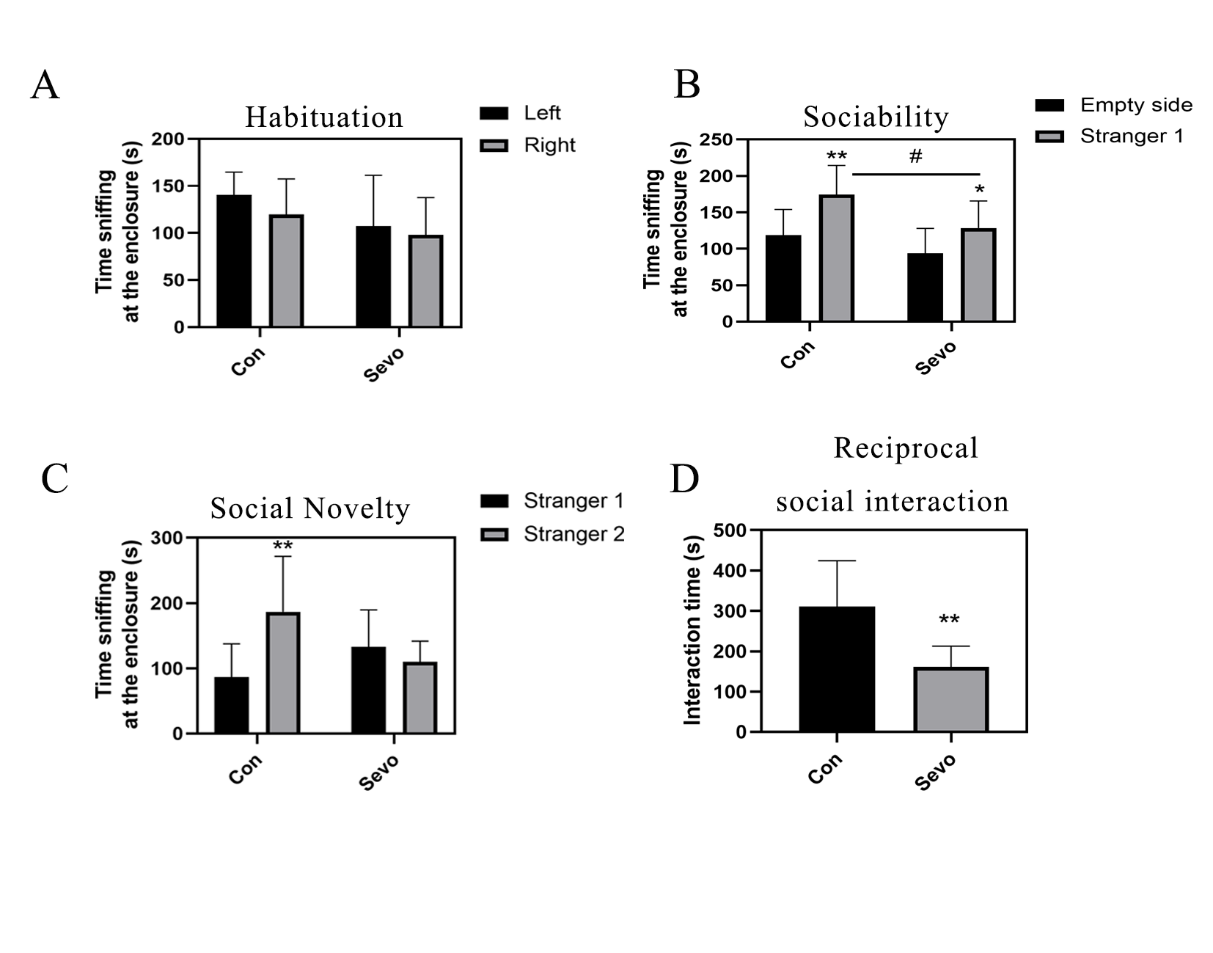


**Footnote**: SEM, standard error of the mean; N, number of mice in each group. Statistical significance was determined using Student’s t-test (*p < 0.05, **p < 0.01, #p < 0.05).

Supplemental Figure 3. Altered Expression of Autism-Related Genes Following Sevoflurane Anesthesia.

(A) mRNA expression of ARID1B. (B) mRNA expression of GABRA5. (C) mRNA expression of GABRB3. (D) mRNA expression of GRIN2B. (E) mRNA expression of SHANK3. (F) mRNA expression of SUV420H1. Values are presented as mean ± SEM. *p < 0.05, **p < 0.01, Student’s t-test. Each group consists of five mice.


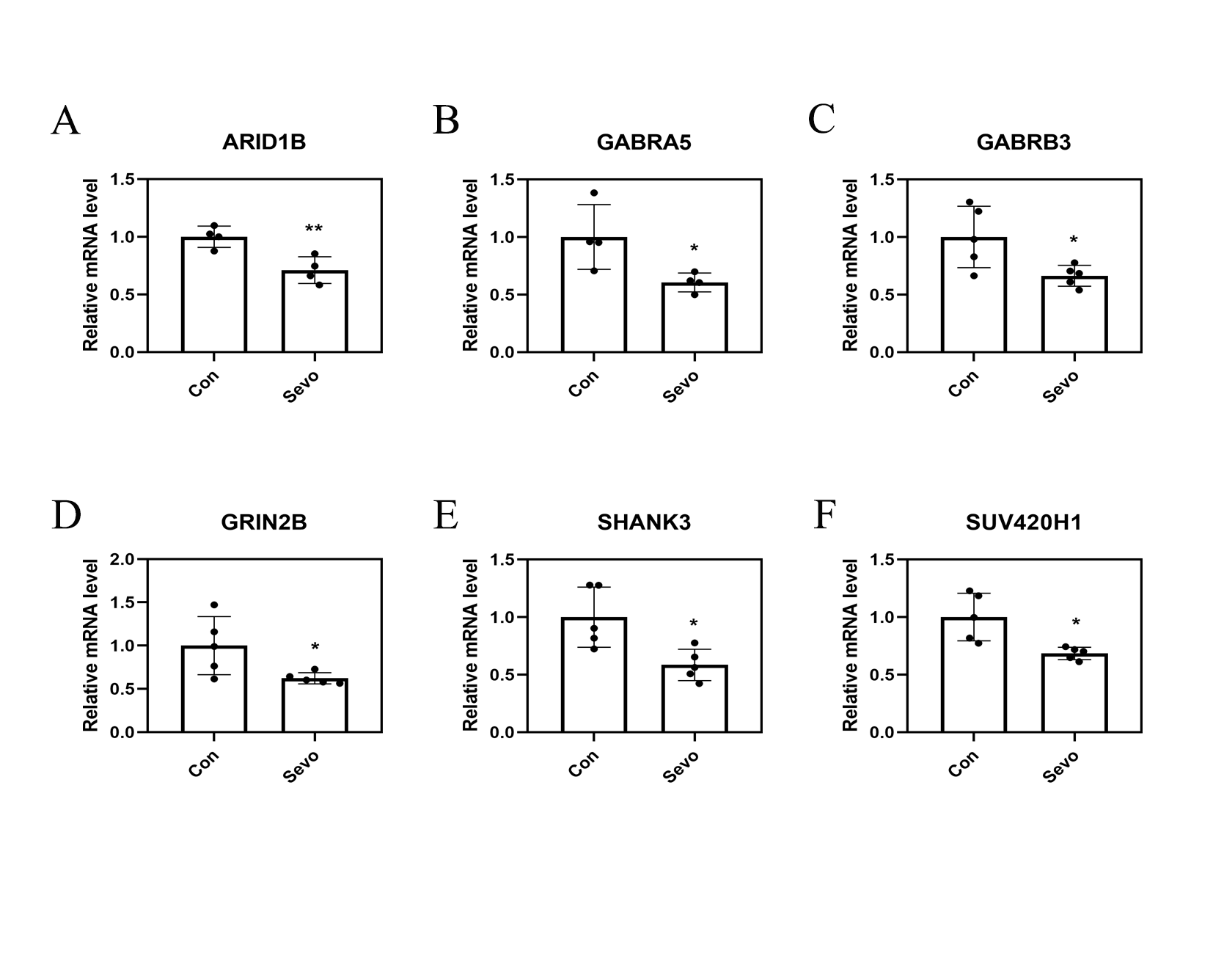


**Footnote**: SEM, standard error of the mean. Statistical significance was determined using Student’s t-test (*p < 0.05, **p < 0.01).
